# Life Cycle of Edible Jellyfish *Acromitus hardenbergi* Stiasny, 1934 (Scyphozoa: Rhizostomeae) Inhabiting a Brackish-Water Environment

**DOI:** 10.3390/ani11072138

**Published:** 2021-07-20

**Authors:** Hiroshi Miyake, Shiho Honda, Jun Nishikawa, Fatimah Md. Yusoff

**Affiliations:** 1 School of Marine Biosciences, Kitasato University, Sagamihara, Kanagawa 252-0373, Japan; mf14018t@st.kitasato-u.ac.jp; 2 Department of Marine Biology, School of Marine Science and Technology, Tokai University, Shimizu, Shizuoka 424-8610, Japan; jun_nishikawa@tokai-u.jp; 3 Department of Aquaculture, Faculty of Agriculture, Putra University Malaysia, Selangor Darul Ehsan, Serdang 43400, Malaysia; fatimamy@upm.edu.my

**Keywords:** *Acromitus hardenbergi*, brackish water, budding, life cycle, mono-disc strobilation

## Abstract

**Simple Summary:**

The edible jellyfish *Acromitus hardenbergi* Stiasny, 1934 is an important fishery resource. The aim of the present study was to elucidate the life history of this brackish-water jellyfish in order to conserve the species and develop sustainable jellyfish fisheries. Matured medusae were collected at the mouth of the Perak River. Primary polyps had a long stalk with a small stolon at the base of the calyx. Fully developed polyps were bowl- or goblet-shaped. Asexual reproduction was accomplished only by means of budding. Strobilation was mono-disc type. Polyps of *A. hardenbergi* expand their population not by podocysts, but by budding as quickly as possible and forming one large ephyra by mono-disc strobilation without the residuum, because the polyp cannot remain for a long time at its settlement place where there is a sediment-rich environment with drastic salinity change.

**Abstract:**

The edible jellyfish *Acromitus hardenbergi* Stiasny, 1934 is harvested throughout the year at the mouth of the Perak River, Malaysia. Although this species is an important fishery resource in the local area, limited biological studies have been carried out on it. The aim of the present study was to elucidate the life cycle of this unique brackish-water jellyfish in order to conserve the species and develop sustainable jellyfish fisheries. Mature medusae were collected at the mouth of the Perak River. Embryonic and larval development after fertilization was completed within 24 h until the planula stage and within 48 h until the polyp stage. Primary polyps had a long stalk with a small stolon at the base of the calyx. Fully developed polyps were bowl-or goblet-shaped but became an elongated stalk under starved conditions. Asexual reproduction was accomplished only by means of budding, and no podocysts were produced. Strobilation was mono-disc type. These characteristics may be adaptations to the dynamic environmental conditions in the estuary of the Perak River, where salinity fluctuates widely due to strong inflows of highly turbid freshwater coupled with tidal changes. This study suggests that polyps of *A. hardenbergi* expand their population not by podocysts, but by budding as quickly as possible and forming one large ephyra by mono-disc strobilation without the residuum, because the polyp cannot remain for a long time at its settlement place in the sediment-rich environment with drastic salinity change.

## 1. Introduction

Jellyfish cuisine is a traditional custom in East Asia. In the 1990s, owing to an increasing demand for jellyfish in Asia, edible jellyfish catches increased to larger than those of scallops or lobsters [1]. Recently, jellyfish demand has drastically increased, leading to expanded jellyfish fisheries worldwide [2,3,4,5]. Southeast Asia is the center of jellyfish exports; exportation of edible rhizostomes from this region account for the majority of the world jellyfish trade [1,6,7,8,9], and they are an important commodity for the fisheries industry in this area. Rhizostome jellyfishes targeted for fisheries in the Southeast Asia are *Crambione mastigophora* Maas, 1903; *Crambionella orsini* (Vanhöffen, 1888); *Crambionella annandalei* Rao, 1931; *Crambionella helmbiru* Nishikawa, Mulyadi and Ohtsuka, 2014; *Lobonemoides robustus* Stiasny, 1920, *Rhopilema esculentum* Kishinouye, 1891; *R. hispidum* (Vanhöffen, 1888); and *Acromitus hardenbergi* Stiasny, 1934 [6,7,10,11]. Jellyfishes are used not only for food but also for fishing bait, fish feed, cosmetics, pharmaceuticals, and material sciences [3], and are also sources of collagen [12,13].

**Figure 1:**
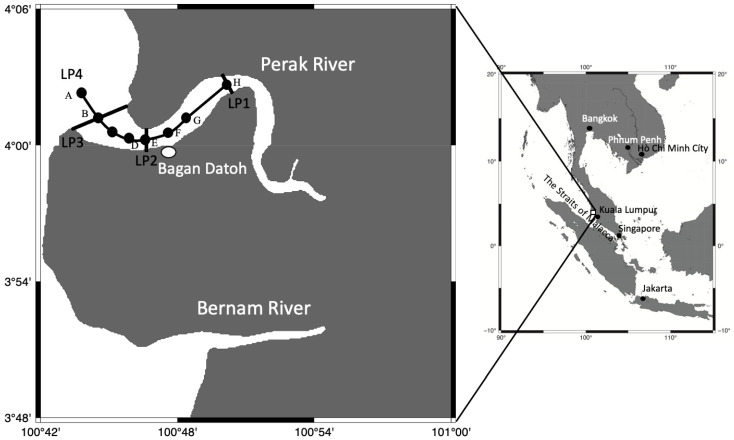
Map of the sampling sites of Perak River in Bagan Datoh, Perak, Malaysia. Black points labelled A–H show CTD casting points. Map was created using GMT 6.0 [17].

In Malaysia, fisheries of *Rhopilema esculentum*, *Rhopilema hispidum*, *Lobonema smithii*, and *Acromitus hardenbergi* are operated in Bagan Datoh, Kukup, and some parts of Sabah and Sarawak [14]. In general, jellyfish fisheries depend on the fluctuations in the temporal and spatial distributions of medusae. On the other hand, fisheries of *A. hardenbergi* are operated all year round in the estuary of Perak River, Malaysia. *Acromitus hardenbergi* is one of the most important jellyfish species for fisheries in this area [10,14]. The Perak is the second longest river (402 km) in peninsular Malaysia and runs into its estuary located at Bagan Datoh to join the Straits of Malacca (Figure 1). The river’s large watershed (14,700 km^2^) provides a wide range of salinity variations in its estuary [15,16]. The Perak estuary is an important area for *Acromitus hardenbergi.*

Recently, it has been suggested that *Acromitus hardenbergi* could potentially be used for the production of nutricosmetics and functional food [12,13]. *Acromitus hardenbergi* will be a commercially and biologically important species in Malaysia. However, jellyfish fisheries in the Perak River are not regulated. Therefore, overfishing of jellyfish and the sustainability of its production are of great concern. Thus, management action plans for sustainable fisheries and conservation of this species are necessary. For the strategic plan to be successful, gaining knowledge of the life cycle of *A. hardenbergi* is required, and should be the first step in establishing a sustainable management plan. However, other than its spatial distribution, limited biological information about *A. hardenbergi* is now available [10,12,14]. As for the life cycle, within the same family, Catostylidae, the life cycle has been described in only three species, *Catostylus mosaicus* (Quoy and Gaimard, 1824) [18], *C. townsendi* Mayer, 1915, and *Acromitoides purpurus* (Mayer, 1910) (personal observations). These species have a similar life cycle to species in Daktyliophorae. Polyps reproduce asexually by podocysts. There is no detailed report on the life cycle of *Acromitus* species. The only report is on polyps of *Acromitus maculosus* [18]. The aim of the present study is to describe the life cycle of this brackish-water jellyfish in order to provide information for the development of sustainable jellyfish fisheries and the conservation of this species.

## 2. Materials and Methods

### 2.1. Environmental Data Collection

Line transect surveys of environmental conditions (water temperature and salinity with depth) were conducted using a CTD (COMPACT-CTD Lite ACTD-CMP, JFE Advantech Co., Ltd., Nishinomiya city, Japan) from the mouth to upstream sites of the Perak Rivers, Malaysia, from 15 June 2015 and 9 January 2016 (Figure 1). The transect lines were placed upstream of the estuary (LP1: 13:33–13:53 on 15 June, 10 km from LP3), at the estuary (LP2:12:04–12:46 on 15 June, 3.5 km from LP3), and on the ocean side of the estuary (LP3: 09:54–10:51 on 15 June) in 2015, and additional stations along the center line of the stream in the estuary (LP4: 11:38–12:52 on 9 January, about 14 km long) were surveyed in 2016 (Figure 1).

### 2.2. Jellyfish Collection

Sampling of *Acromitus hardenbergi* was conducted in estuaries of the Perak River on 3–4 October 2012, 20–21 September 2013, 27–28 December 2014, 13–16 June 2015 and 8–11 January 2016. The sampling was conducted with permission under JSPS cooperative projects and academic exchange contracts.

Medusae were collected at the river mouth (between LP2 and LP3 in Figure 1) with a small set-net (100 cm in diameter, 2 mm mesh), a fine-meshed rectangular dip-net (30–21 cm, about 200–300 µm mesh (Super Color Net, Kamihata Fish Industries Ltd. Shiroganemachi Himeji, Japan) or a ladle (20 cm in diameter) from a boat, and they were kept in a tub on the boat. The bell diameter (mm) and wet weight (g) of the collected medusae were measured immediately after landing, and their maturity was checked based on the presence or absence of gonads. *Acromitus hardenbergi* medusae used for the fertilization experiment were collected on the last day of sampling in the area around the river mouth where the temperature was 31–32 °C and salinity was 8–12.

**Figure 2:**
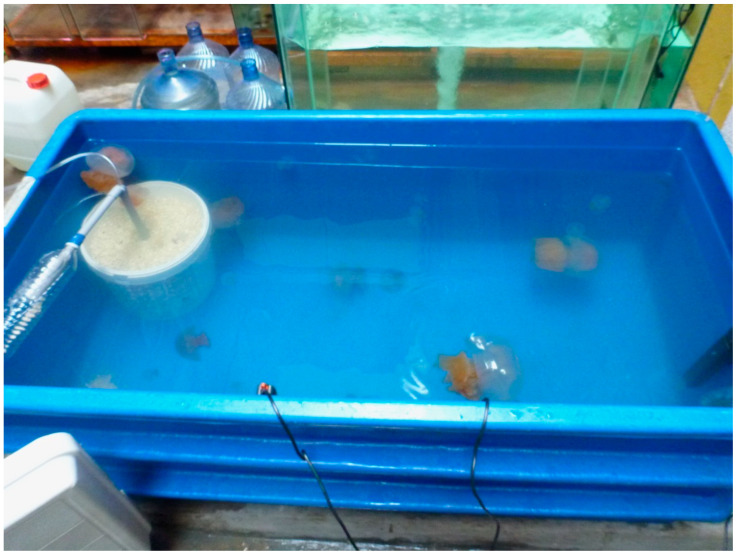
Medusae of *Acromitus hardenbergi* kept in an FRP tank.

Intact adult medusae of *A. hardenbergi* collected from the Perak River were individually packed in plastic bags with oxygen tablets [19] and kept in foam cooler boxes during transport to the Institute of Bioscience, Universiti Putra Malaysia (UPM). Medusae (*n* = 15) were transferred to a fiber-reinforced plastic (FRP) tank (500 L) with an undergravel filter set in a bucket (Figure 2). The tank was prepared prior to the sampling trip at the outdoor aquarium space of UPM. The rearing seawater was maintained at 30 °C and salinity of 15 based on habitat water temperature and salinity from a previous survey [16]. The light condition was not controlled and depended on sunrise and sunset. Fertilized eggs with polar bodies and two-cell-stage embryos were collected by filtering the rearing water using a plankton net (50 µm mesh) set in the tank from 09:30 to 17:00 on the day after medusae were released into the tank. The collected fertilized eggs and embryos were kept in plastic Petri dishes (78 mm in diameter, 24 mm in height) filled with natural seawater (salinity 15) at room temperature (approximately 25 °C) for observation of development into planulae. In addition, many planulae were collected using the plankton net from the rearing tank the next day when the Japanese team returned to Japan. The planulae were kept alive in plastic Petri dishes packed in a foam cooler box and brought to the laboratory at Kitasato University, Japan.

### 2.3. Animal Maintenance

The plastic Petri dishes (78 mm in diameter, 24 mm in height) containing the planulae were kept in an incubator at 30 °C, which was the water temperature of their habitat, until settlement as polyps. The polyps were kept in the Petri dishes filled with filtered natural seawater (1.0 µm) at a salinity of 10 and temperature of 30 °C. The salinity and temperature were derived from measured results. The light condition was not regulated. The incubator had a window to check inside. Only room lighting shone into the incubator from about 09:00 to 19:00. Rotifer, *Brachionus plicatilis* sp. complex, and *Artemia* nauplii were fed to the polyps every other day. Polyps were fed to satiation beyond what they could eat. *Artemia* nauplii were chopped up using a microfine needle under a stereomicroscope (SZX 10, Olympus) and fed directly to newly developed polyps using the microfine needle. The rearing water was completely replaced with filtered seawater (1.0 µm) under the same temperature and salinity conditions a few hours after feeding. When the polyps released ephyrae after the strobilation, they were cultured in a plastic bottle (1 L) filled with filtered water (1.0 µm) at a salinity of 15 and temperature of 30 °C with circulation by aeration (5 bubbles/s). The metaephyrae and medusae were kept in a drum-shaped aquarium (4 L, 21 cm in diameter, 13 cm wide) at a salinity of 15 and temperature of 30 °C with circulation by aeration (5 bubbles/s). *Artemia* nauplii were fed to the ephyrae, metaephyrae, and medusae beyond what they could eat every other day. The rearing water was completely replaced with filtered water (1.0 µm) under the same conditions a few hours after the feeding. The animal experiment in this study was approved by the animal experiment ethics committee of the School of Marine Biosciences, Kitasato University, with approval identification code MB180510b.

### 2.4. Examination of Suitable Salinity for Planula Settlement

At Kitasato University, the optimal salinity for planula settlement was examined at a temperature of 30 °C and salinity of 3, 5, 10, 15, 20, and 30, which mimicked the salinity range (0–20) in the Perak River estuary [15,16] and in normal seawater (30). A batch of 10 planulae 3 days after the fertilization was introduced into one well of a 12-well microplate filled with 5 mL of seawater with each salinity level. The replicates consisted of 6 wells for each salinity level. After 7 days, the numbers of settled polyps and unsettled planulae were counted under a stereomicroscope.

### 2.5. Observation of Life Cycle

Observations of early development from fertilized eggs to planulae using a stereomicroscope were conducted at 2, 4, 6, and 24 h after the collection of fertilized eggs. Morphological observations of polyps, strobilae, ephyrae, metaephyrae, and medusae were similarly conducted, and when animals relaxed under the microscope, images were taken from directly above using a digital camera (EOS Kiss X6i, Canon, Tokyo, Japan) mounted on the stereomicroscope or biological microscope. The mouth disc diameter of the polyps and strobilae and the central disc diameters of the ephyrae [20] and medusae were measured from photo images using Image J software [21].

### 2.6. Statistical Analysis

One-way ANOVA was performed for the planula settlement experiment to compare the settlement and mortality rates of planulae at different salinity levels. ANCOVA was performed for the allometric relationship between medusa size and wet weight in the 2015 and 2016 populations. The software for the statistical analysis was Prism 8 for Mac v8.4.0 (GraphPad Software, San Diego, CA, USA).

## 3. Results

### 3.1. Environmental Conditions in the Perak River

The water of the Perak River was highly turbid (visibility: 20 cm at most), and the bottom was muddy. During high tides, strong tidal currents swept upstream, and the jellyfish drifted with the tide to upstream areas. There were mangrove trees around the estuaries and palm trees on both sides of the riverbanks in the upstream area. Many fine fragments of palm materials drifted in the water and were collected by the jellyfish set nets. At low tide, vast tidal flats emerged at the river mouth.

**Figure 3:**
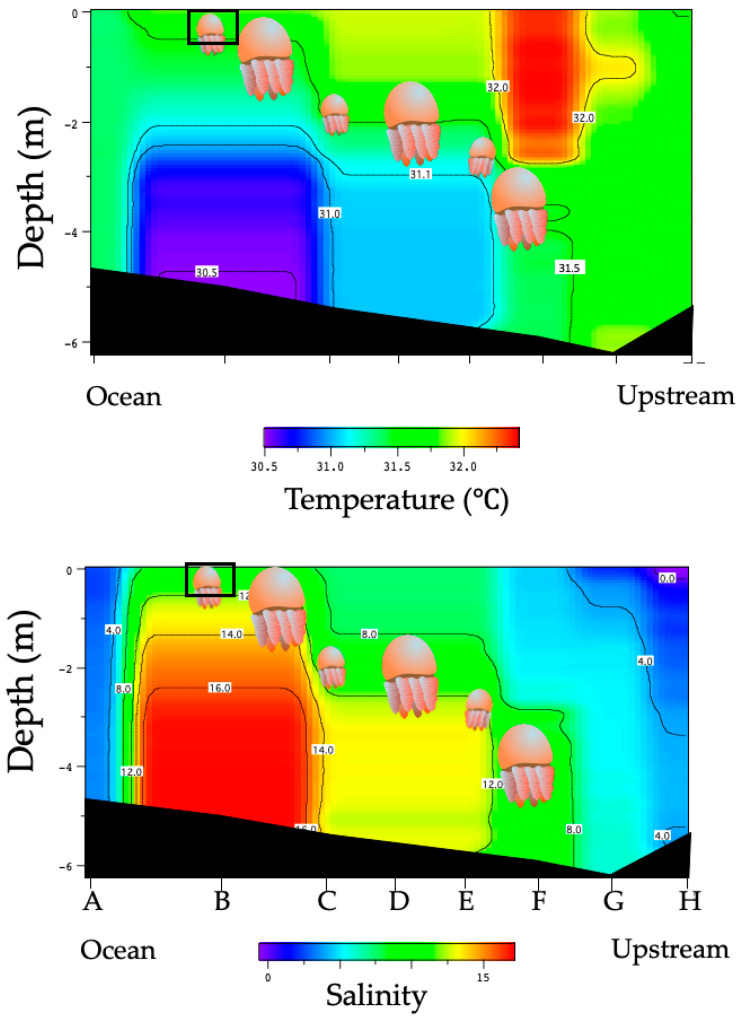
Profiles of temperature and salinity along LP4 in Perak River at low tide. Letters indicate places where CTD was cast, as shown in Figure 1. Black rectangle shows occurrence area of medusae along this transect survey. Possible jellyfish distribution in this survey is shown with jellyfish images.

In the river, temperature and salinity were 29.1–29.3 °C and 0.1–0.5 at the upstream line (LP1), 29.0–29.3 °C and 0.5–3.5 at the estuary line (LP2), and 28.7–29.2 °C and 2.0–10.0 at the ocean-side line (LP3) (Table 1). The environmental characteristics from upstream to the ocean (LP4) are shown in Figure 3. The temperature and salinity of LP4 were 31.5–32.0 °C and 0.0–4.0 at the upstream line, 30.5–31.5 °C and 8.0–12.0 at the estuary line, and 30.5–31.5 °C and 8.0–16.0 at the ocean side line, respectively (Figure 3).

### 3.2. Distribution of Acromitus hardenbergi in the Perak River

**Figure 4:**
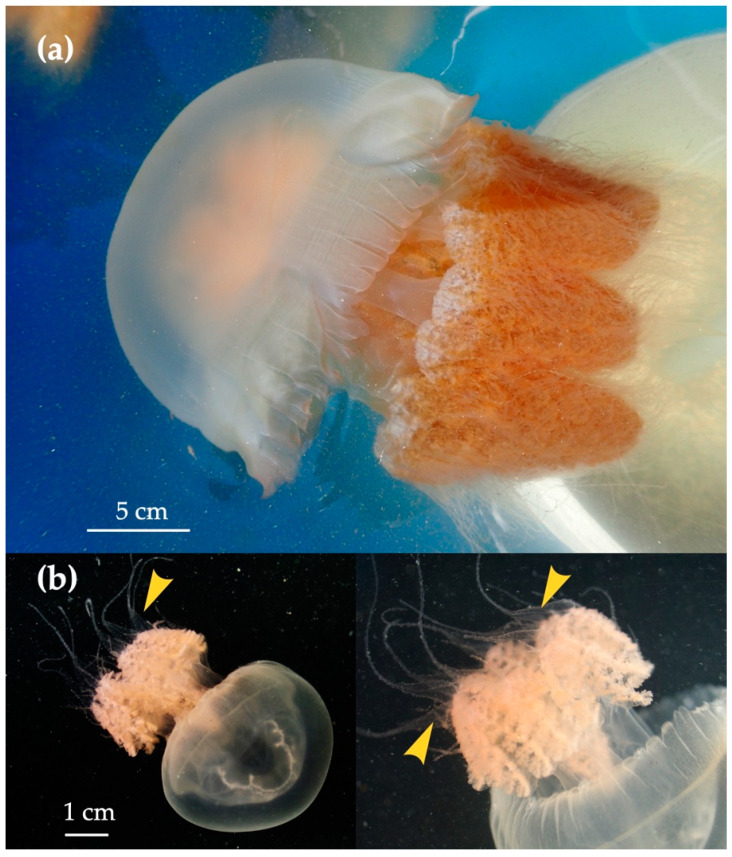
*Acromitus hardenbergi* Stiasny, 1934: (**a**) adult medusa; (**b**) appendage (arrow).

*Acromitus hardenbergi* was found on every survey trip. The medusae of *A. hardenbergi* that were collected carefully by ladle had whip-like appendages (Figure 4), but the individuals collected by dip net and a set net lost them. *Acromitus hardenbergi* was found in all areas from LP1 to LP3 (Figure 1), but was not observed in the ocean and upstream areas from LP1, where palm trees were observed on both sides of the riverbank. *Acromitus hardenbergi* was also observed in the surface layer (black rectangle in Figure 3) with salinity of 8–12 around the estuary along the LP4 transect line.

### 3.3. Seasonal Difference in Size Distribution of A. hardenbergi Medusae in Perak River

**Figure 5:**
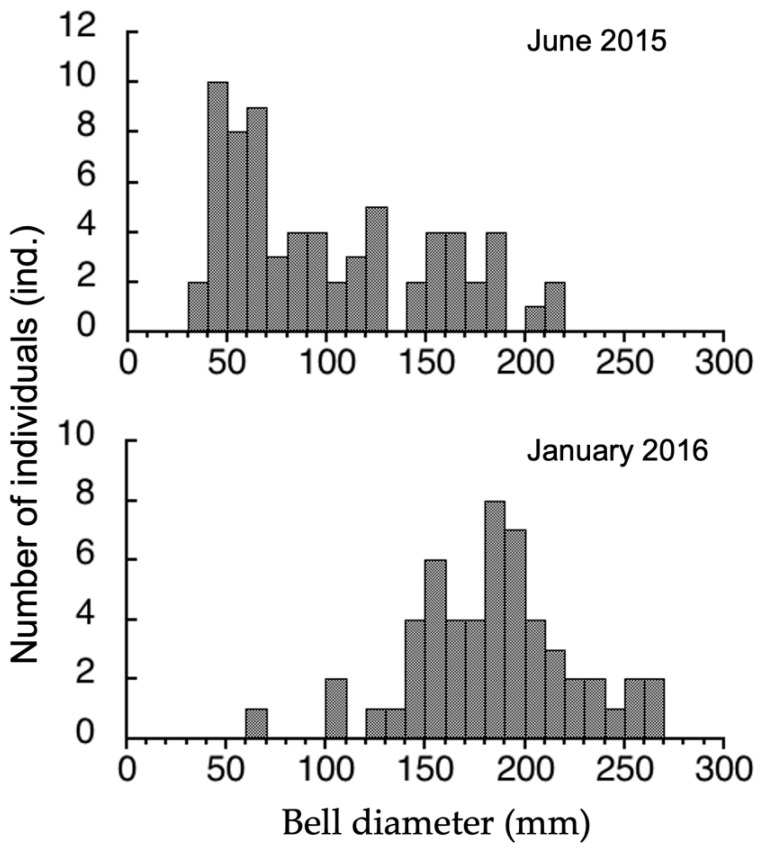
Size distribution of *Acromitus hardenbergi* medusae collected in June 2015 and January 2016.

**Figure 6:**
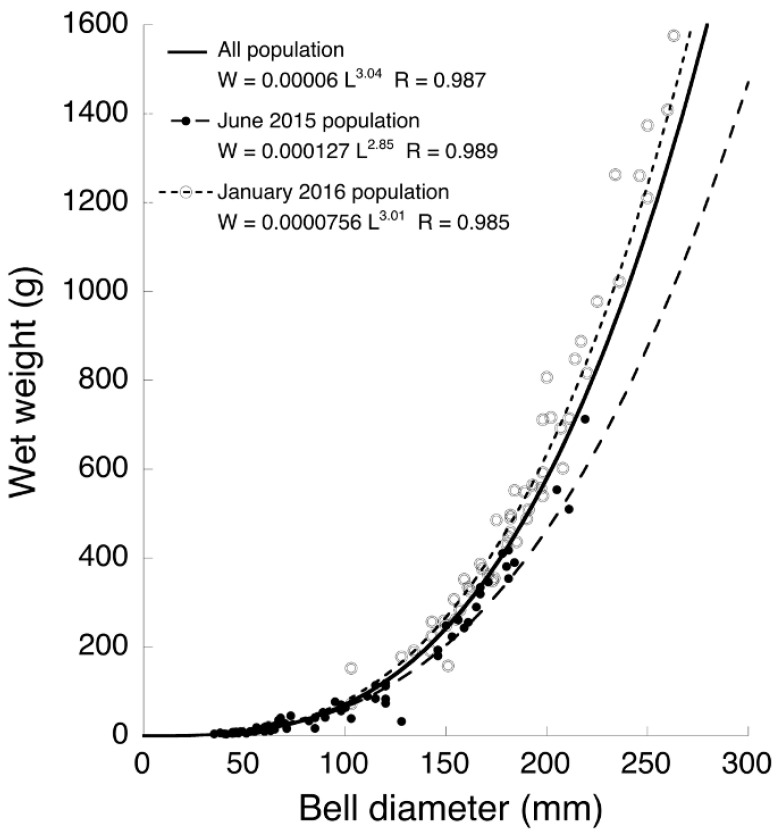
Relationship between bell diameter and wet weight of *Acromitus hardenbergi* medusae collected in different sampling periods.

Only two medusae (10 and 24 mm) were collected in December 2014 because there was an unprecedented flood in the upper stream of the Perak River. The bell sizes and wet weights of all medusae collected by the set nets in 2015 (*n* = 69) and 2016 (*n* = 54) were measured. In June 2015, the bell diameter ranged from 35 to 219 mm (99.9 ± 51.7 mm on average ± standard deviation (SD)) (Figure 5) and the wet weight ranged from 3.7 to 713 g. Most of the medusae (55.0%) were 40–100 mm and did not mature in June 2015. In January 2016, the bell diameter was 62–289 mm (182.5 ± 40.1 mm on average ± SD) (Figure 5) and the wet weight was 19.9–1576 g. Most of the medusae (53.7%) were 150–200 mm and mature. The relationship between medusa size (L, mm) and wet weight (W, g) is expressed by W = 0.00006 *L*^3.04^ (R = 0.987) for the whole population, *W* = 0.000127 *L*^2.85^ (R = 0.989) for the June 2015 population, and W = 0.0000756 *L*^3.01^ (R = 0.985) for the January 2016 population (Figure 6).

### 3.4. Life Cycle of Acromitus hardenbergi

The large medusae spawned in the FRP tank in which they were maintained. Planulae were successfully collected from mature medusae in October 2012, September 2013, and January 2016, but no planulae were obtained in June 2015 because of the immaturity of collected medusae.

All of the medusae brought back to UPM were alive and were placed in a rearing FRP tank allowing for potential sexual reproduction as soon as possible. Fertilized eggs and two-cell-stage embryos were collected the next morning, but no more fertilized eggs were seen in the afternoon and at night. Fertilization of *A. hardenbergi* occurs externally. *Acromitus hardenbergi* has no brooding sacs around its oral arms, and planulae were not observed inside the gonadal cavity or on the oral arms of collected medusae.

**Figure 7:**
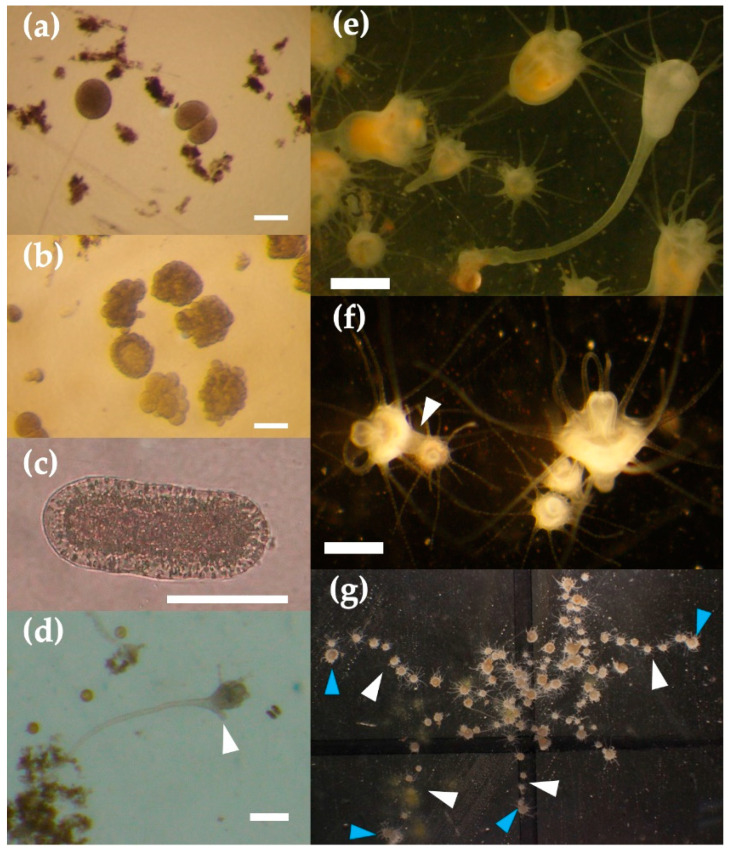
Life stages of *Acromitus hardenbergi* (egg to polyp). (**a**) Fertilized egg and two-cell embryo stage, scale bar = 100 µm; (**b**) multi-cell and blastocyst embryos stages, scale bar = 100 µm; (**c**) planula, scale bar = 100 µm; (**d**) primary polyp with short stolon just under calyx (white arrow), scale bar = 200 µm; (**e**) fully developed polyps and long-stalked tulip-type polyp (white arrow), scale bar = 500 µm; (**f**) asexual reproduction by budding (white arrow), scale bar = 500 µm; (**g**) clonal colony, where blue arrows show the mother polyps and white arrows show line of reproduced daughter polyps.

Embryos had negative buoyancy and were about 113.4 ± 13.5 µm in diameter, on average. The fertilized eggs and two-cell-stage embryos collected at 09:30 passed through the 32- or 64-cell stages within two hours (Figure 7a). The fertilized eggs developed into blastulae in 6 h and into planulae within 24 h after fertilization (Figure 7b). The external morphology of the planulae was pear-shaped, and the average size was 168.8 ± 25.3 µm along the long axis (Figure 7c). The planulae attached to the bottom of the plastic Petri dishes, sand granules, or large suspended particles in the rearing water within two days after fertilization.

The primary polyps had a wineglass shape with a long stalk and a short stolon just under the calyx and two to six tentacles until about 18 days after settlement (Figure 7d). The average mouth disc diameter was 185 ± 42 µm (*n* = 12). The fully-developed polyps 19 days after settlement were bowl- or goblet-shaped with, generally, 16 tentacles (range 13–18), and the average mouth disc diameter was 387.3 ± 106.8 µm (*n* = 45) (Figure 7e). When food was limited because of an accident in *Artemia* hatching, the starved polyps elongated their stalks (Figure 7e). The attachment area of the pedal disc was small. Asexual reproduction was conducted by typical lateral budding [22,23] and no podocysts were observed (Figure 7f). The bud was formed at the lower part of the calyx and developed into an eight-tentacled polyp within one or two days from budding. The mother polyp formed a new bud within one day after the daughter polyp, which had 8 or 16 tentacles, detached from the mother polyp. The polyps were arranged in a line in the clonal colony because the parental polyps moved linearly to another place, leaving daughter polyps behind where they detached from the mother polyp. (Figure 7g).

**Figure 8:**
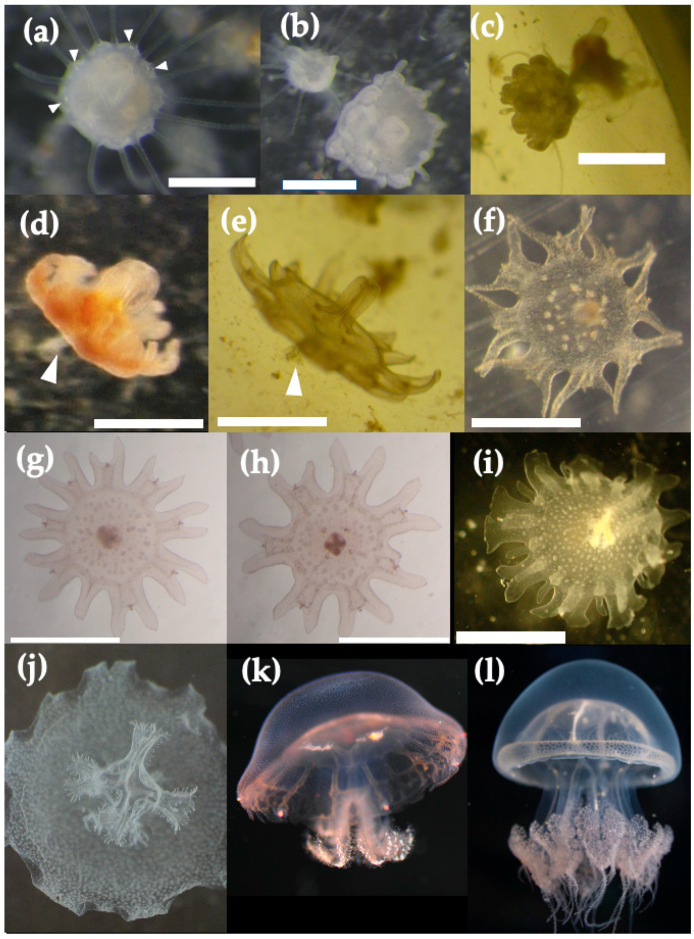
Life stages of *Acromitus hardenbergi* (strobila to medusa): (**a**) primary stage of strobilation, white arrows show statoliths, scale bar = 500 µm; (**b**) intermediate stage of strobilation, scale bar = 500 µm; (**c**) late stage of strobilation, scale bar = 1 mm; (**d**,**e**) final stage of strobilation, white arrows show tiny residuum, scale bar = 500 µm; (**f**–**h**) ephyra, scale bar = 1 mm; (**i**) ephyra (7 days), scale bar = 2 mm; (**j**) metaephyra (20 days); (**k**) juvenile medusa (2 months); (**l**) young medusa. (**a**–**k**) Breeding individuals, (**l**): wild individual.

The first strobilation was observed 14 days after the settlement of the planulae under rearing conditions. Mono-disc strobilation was initiated when the food supply was limited because of an accident in *Artemia* hatching (Figure 8a–e). Under regular feeding conditions at intervals of two days, polyps did not strobilate. At the beginning of strobilation, statoliths were formed at the base of every two tentacles (Figure 8a, white arrows). Then the calyx body of polyps became short and flat, and eight primordia of ephyral lappet were formed in a day (Figure 8b,c). Tentacles at the primordia of ephyral lappet disappeared with the development of the lappet, followed by the development of marginal lappets, when other tentacles between marginal lappets disappeared with ephyra formation (Figure 8c). Most body parts of polyps transformed to ephyra except for the bottom part of the stalk (Figure 8d,e). The strobilae released ephyrae (1.1 ± 0.15 mm, *n* = 9) within one or two days after the initiation of strobilation. The tiny residuum (Figure 8d,e, white arrows) disappeared without regenerating into the polyp after liberation of an ephyra.

Ephyrae had eight rhopalia with lancet-like rhopalial lappets. The number of rhopalia ranged from 4 to 10. Nematocyst warts were distributed on the central discs of the ephyrae; some were totally scattered on the center part of the discs, whereas others were scattered on the inner halves of the discs (Figure 8f–h). The ephyrae grew from 1.1 to 2.2 mm over one week (Figure 8i) and developed into metaephyrae in 10–20 days (Figure 8j). In the metaephyra stage, the manubrium elongated and branched to developed into oral arms with many small mouths (Figure 8j). The ephyrae developed into juvenile medusae one month after liberation (Figure 8k).

### 3.5. Suitable Salinity for Planula Settlement

**Figure 9:**
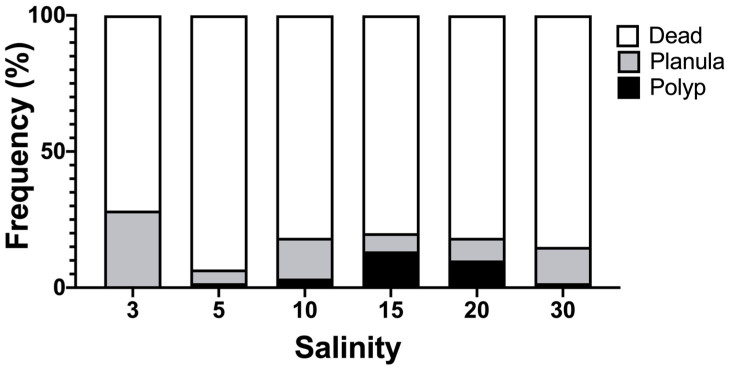
Frequency of settled, swimming and dead planulae at different 6 salinity levels over 7 days.

The settlement and development of *A. hardenbergi* planulae depended on salinity (Figure 9). The average settlement rates (*n* = 6 replicates) of planulae over 7 days were 0.00 ± 0.00%, 1.67 ± 4.08%, 3.33 ± 5.16%, 13.33 ± 10.33%, 10.00 ± 8.94%, and 1.67 ± 4.08% at salinity of 3, 5, 10, 15, 20, and 30, respectively. Settlement rates were significantly higher at salinity of 15 and 20 (one-way ANOVA followed by post hoc Fisher’s LSD test: F (5, 30) = 4.216, *p* = 0.0051) The percentage of planulae swimming at the end of the experiments was 28.33 ± 34.30%, 5.00 ± 12.25%, 15.00 ± 17.61%, 6.67 ± 10.33%, 8.33 ± 9.83%, and 13.33 ± 10.33% at salinity of 3, 5, 10, 15, 20, and 30, respectively. There were significant differences between the salinity of 3 and 5, and 3 and 15 (one-way ANOVA followed by post hoc Fisher’s LSD test: F (5, 30) = 1.349, *p* = 0.2711). The mortality rates were 71.67 ± 34.30%, 93.33 ± 12.11%, 81.67 ± 19.41%, 80.00 ± 8.94%, 81.67 ± 14.72%, and 85.00 ± 10.49% at salinity of 3, 5, 10, 15, 20, and 30, respectively. There was no significant difference (one-way ANOVA followed by post hoc Fisher’s LSD test: F (5, 30) = 0.8481, *p* = 0.5268).

## 4. Discussion

### 4.1. Medusa Stage

Allometric growth of medusae was different between the January and June populations (Figure 6). Wet weight relative to bell diameter was significantly higher in the January 2016 than the June 2015 population (ANCOVA: *p* < 0.0001). Jellyfish can easily change their body size in response to food volume availability [24,25,26]. The mean density of zooplankton at the estuary of the Perak River in the Northeast (NE) monsoon season was low compared with the upstream and marine areas because of the high predation pressure by *A. hardenbergi* [15]. On the other hand, zooplankton abundance at the estuary was about 6–70 times higher in January than in the other months of the NE monsoon season, because of the high inflow of zooplankton with freshwater from upstream due to high precipitation [15]. The heavy wet weight of the January population could have resulted from the food-rich environment.

Many large, mature *A. hardenbergi* were collected in September 2013, October 2012, and January 2016, but mostly small individuals and a few mature ones were collected in June. This suggests that the breeding season of *A. hardenbergi* in the Perak River occurs from September to January. Spawning occurred only in the morning, suggesting that it was triggered by the transition from dark to light conditions, as reported for *Nemopilema nomurai* Kishinouye, 1922 [27], *Cyanea nozakii* Kishinouye, 1891 [28], *Cytaeis uchidae* Rees, 1962 [29], *Aurelia coerulea* von Lendenfeld, 1884, and *Rhopilema asamushi* Uchida, 1927 (personal observations). *Acromitus hardenbergi* may spawn in the morning to increase fertilization efficiency. The species was observed in the surface layer with salinity 8–12 around the estuary, as shown in Figure 3. The jellyfish swimming area was limited by pycnocline, density, or salinity [30,31,32]. *Acromitus hardenbergi* can be distributed in the layer with salinity of 8–12 on the saltwater wedge and be pushed up and aggregated in the surface layer by the saltwater wedge and inflow of freshwater. If this phenomenon occurs in the morning, it may play a great role in the efficiency of the fertilization process triggered by the transition from dark to light around sunrise time.

Strobilation was observed in the laboratory when the food supply was limited, without water temperature or salinity change. On the other hand, in Kamo Aquarium, Yamagata, Japan, strobilation occurred when salinity increased from 10 to 20 and decreased from 20 to 10 without food depletion (Okuizumi and Ikeda, personal observation). Zulikha et al. [15] reported that zooplankton abundance was lower at the Perak River estuary in the NE monsoon season from November to February. It was suggested that the low zooplankton abundance was due to high predation pressure by the *A. hardenbergi* population [15]. Drastic salinity changes also occurred due to the large inflow of freshwater from upstream by the high precipitation in the monsoon season [15]. Temperature shift is a strobilation stimulus in many species [33], however most of them are temperate to cold species. Tropical regions have little change in water temperature during the year. Some species need salinity shifts for strobilation [33] Although future experimentation is needed, the stimulus or acceleration factors for strobilation are presumed to be food depletion and salinity change. The NE monsoon season may be important for *A. hardenbergi* breeding and strobilation. In this study, strobilae were observed at least two weeks from planulae settlement and ephyrae were released in one day. The ephyrae grew to young medusae in two months. Small medusae (10 and 24 mm) were also collected in December. Many medusae that could reproduce were observed in January (Figure 5). These facts suggested that the ephyrae released in the NE monsoon season were recruited into the population as a new generation and grew to medusae in June (Figure 5). This system may allow fishermen in Bagan Datoh to fish *A. hardenbergi* throughout the year, as reported by Nishikawa et al. [10] and Yusoff et al. [14].

### 4.2. Planula Stage

The swimming period until settlement for planulae was shown to be 4–5 day in *Catostylus mosaicus* (Quoy and Gaimard, 1824) [18], 4–8 day in *Nemopilema nomurai* [34], within 4 day in *Rhopilema esculentum* [35], 2–5 day in *Stomolophus meleagris* Agassiz, 1862 [36], 3–5 day in *Rhizostoma luteum* (Quoy and Gaimard, 1827) [37], and 1–4 day in *Lychnorhiza lucerna* Haeckel, 1880, which inhabits an estuary with salinity of 17 [38]. The swimming period of planulae in *A. hardenbergi* is relatively short and can be as short as one day. Schiariti et al. [38] reported that planulae of *Lychnorhiza lucerna* inhabiting brackish water settled on substrate within at least one day. In this study, *A. hardenbergi* planulae were able to attach to hard substrates, such as the bottom of Petri dishes, and soft substrates, such as macro-suspended matter (e.g., fragments of palm fruit skin). The short swimming period of *A. hardenbergi* planulae and their ability to settle on soft substrates may be a survival strategy for the harsh environment of the estuary, where the water flows fast, and the planulae are subjected to drastic changes in salinity and muddy water with much macro-suspended matter.

The results of the settlement rates in different salinity levels suggest that the optimal environment for planula settlement is salinity of 15–20, which corresponds to the preferable salinity range of the polyps. This range is relatively narrow. However, it is important for the survival of polyps, because attached planulae in the estuary provide the polyps with a suitable saline environment without exposing them to extremely low salinity by tidal action. The high mortality rate at different salinity levels may be due to transportation from Malaysia to Japan. It took some time before we could start the settlement experiment in Japan, and it is possible that it was not the best time for planula settlement.

The mortality rate of planula was 82.2 % on average, which is a high rate compared with other scyphozoans [39]. Planulae used in this experiment were 3-day-old individuals after fertilization. The planulae of *A. hardenbergi* have a relatively short swimming period, and the planulae used in this experiment were possibly too old for the settlement experiment. The polyps were kept healthy and ephyrae were released in a salinity of 15. Polyps may inhabit environments with low salinity (around 15). Toyokawa (2011) found polyps of *Chrysaora pacifica* (Goette, 1886) on shells and stones at the sea bottom shallower than 5 m depth in Sagami Bay near the mouth of Sagami River. We could not find polyps in the field; polyps of *A. hardenbergi* can be found on shells or stones at the estuary bottom with salinity around 15.

### 4.3. Polyp Stage

The polyps were normally bowl- or goblet-shaped, but young or starved polyps had a tulip-like shape with an elongated stalk. Long-stalked tulip-type polyps have also been observed in *Cassiopea* spp. and *Cotylorhiza tuberculata* (Macri 1778) in Scyphozoa and *Malo maxima* Gershwin, 2005 and *Morbakka virulenta* (Kishinouea, 1910) in Cubozoa [40,41,42,43]. A long stalk allows these polyps to cover a wider range of the water column for feeding [43]. The long-stalked tulip-shaped polyps had a small projected stolon just under the calyx. In addition, the long stalk is an adaptation to constant sedimentation, since polyps that inhabit heavily sedimented bottoms, like *M. virulenta*, also have a long stalk [42]. The Perak River is filled with heavily muddy water with little transparency, and suspended silt and fine fragments of palm trees and fruits are abundant. The behavior of *A. hardenbergi* polyps was similar to that of polyp of *Sanderia malayensis* Goette, 1886, which were found on the tube of a vestimentiferan tubeworm, *Lamellibrachia satsuma* Miura, Tsukahara and Hashimoto, 1997 in Kagoshima Bay [44]. The *S. malayensis* polyp can move three-dimensionally from tube to tube in tubeworm colonies by using the tips of its stolon. The area of the pedal disc of *A. hardenbergi* polyps is small, allowing them to detach from and reattach to the substrate quickly. *Acromitus hardenbergi* polyps also have a long stalk with a small projected stolon. There are mangroves, a sandy bottom, and large amount of suspended matter in the Perak River estuary, which can serve as attachment substrates for polyps or planulae. *Acromitus hardenbergi* polyps can move three-dimensionally on those substrates using their morphological features.

The podocysts of *Aurelia* endure harsh environments when perpetuating the polyp population and excysts when the environment improves [23,45,46]. However, the population growth rate by podocyst production is considerably slower than that by budding [23]. Although further studies are needed to confirm other modes of asexual reproduction due to other environmental conditions, one reproductive strategy of *Acromitus hardenbergi* seems to be that the polyps expand their population by budding as quickly as possible in the river mouth where the water has a fast flow, with drastic changes in sediment loads and salinity levels.

### 4.4. Strobilation Stage

The size of fully-developed *A. hardenbergi* polyps was about 0.4 mm, which is smaller compared to other scyphistomae. Therefore, *A. hardenbergi* polyps made ephyrae as large as possible by mono-disc strobilation and tiny residuum tissue remained, like the direct development of planula (planula strobilation) in *Aurelia coerulea* [47,48,49,50]. However, the residuum could not develop into the polyp, and it disappeared. The strobilation strategy of *A. hardenbergi* seems to be that the polyp does not stay at its settlement place in the sediment-rich environment with drastic salinity change after strobilation without the residuum. This strategy may increase the ephyra survival rate by providing the ephyra with most of the polyp’s energy and nutrients. To verify the hypothesis of this strobilation strategy, further experimentation should be done in terms of the bioenergetic aspects.

## 5. Conclusions

Our results suggest that *A. hardenbergi* has adapted to an estuarine environment consisting of drastic salinity changes, abundant sediment, and a fast current. Adult medusae exist and may breed in the Perak River all year round. Breeding may occur mainly in the NE monsoon season. The efficiency of fertilization is accelerated when jellyfishes are aggregated by the seawater wedge and the inflow of freshwater in the early morning. Planulae quickly attach to the substrate where salinity does not become too low. The polyp size is small, but the polyps grow colonies as fast as possible by budding. Despite the small size of some polyps, mono-disc strobilation from the whole body of the polyp maximizes the size of newly released ephyrae, which positively affects survival. Strobilation may occur all year round. The main season of strobilation may be the NE monsoon season, because the stimuli for strobilation are food depletion and salinity change. This life cycle strategy has putatively allowed *A. hardenbergi* medusae to occur throughout the year in the Perak River. The jellyfish fisheries in the Perak River can also be operated all year round.

## Figures and Tables

**Table 1:** Temperature and salinity at survey sites in Perak River.

Transect Line	Temperature (°C)	Salinity	Distance from LP3
LP1	29.1–29.3	0.1–0.5	10 km
LP2	29.0–29.3	0.5–3.5	3.5 km
LP3	28.7–29.2	2.0–10.0	0

The transect lines LP1, 2, and 3 were showed in Figure 1.

## Data Availability

All data sets collected and analyzed during the current study are available from the corresponding author on fair request.
